# The complete chloroplast genome sequence of Asian Palmyra palm *(Borassus flabellifer)*

**DOI:** 10.1186/s13104-017-3077-8

**Published:** 2017-12-16

**Authors:** Arpakorn Sakulsathaporn, Passorn Wonnapinij, Supachai Vuttipongchaikij, Somsak Apisitwanich

**Affiliations:** 1 Center for Agricultural Biotechnology, Kasetsart University, Kamphaeng Saen Campus, Nakhon Pathom, 73140 Thailand; 2 Center of Excellence on Agricultural Biotechnology: (AG-BIO/PERDO-CHE), Kasetsart University, 50 Ngarm Wong Wan Road, Chattuchak, Bangkok, 10900 Thailand; 3 Department of Genetics, Faculty of Science, Kasetsart University, 50 Ngarm Wong Wan Road, Chattuchak, Bangkok, 10900 Thailand; 4 Center of Advanced Studies for Tropical Natural Resources, Kasetsart University, 50 Ngam Wong Wan, Chattuchak, Bangkok, 10900 Thailand; 5 Special Research Unit in Microalgal Molecular Genetics and Functional Genomics (MMGFG), Department of Genetics, Faculty of Science, Kasetsart University, 50 Ngarm Wong Wan Road, Chattuchak, Bangkok, 10900 Thailand; 6 School of Science, Mae Fah Luang University, Chiang-Rai, 57100 Thailand

**Keywords:** Arecaceae, Borasseae, Commelinids, Phylogeny, Plastid, Tandem repeats

## Abstract

**Objective:**

*Borassus flabellifer* or Asian Palmyra palm is widely distributed in South and Southeast Asia and is horticultural and economic importance for its fruit and palm sugar production. However, its population is in rapid decline, and only a few genetic data are available. We sequenced the complete chloroplast (cp) genome of *B. flabellifer* to provide its genetic data for further utilization.

**Results:**

The cp genome was obtained by Illumina sequencing and manual gap fillings providing 160,021 bp in length containing a pair of inverted repeats (IRs) with 27,256 bp. These IRs divide the genome into a large single copy region 87,444 bp and a small single copy region 18,065 bp. In total, 113 unique genes, 134 SSRs and 47 large repeats were identified. This is the first complete cp genome reported in the genus *Borassus.* A comparative analysis among members of the Borasseae tribe revealed that the *B. flabellifer* cp genome is, so far, the largest and the cp genomes of this tribe have a similar structure, gene number and gene arrangement. A phylogenetic tree reconstructed based on 74 protein-coding genes from 70 monocots demonstrates short branch lengths indicating slow evolutionary rates of cp genomes in family Arecaceae.

**Electronic supplementary material:**

The online version of this article (10.1186/s13104-017-3077-8) contains supplementary material, which is available to authorized users.

## Introduction

*Borassus flabellifer* or Asian palmary palm (family Arecaceae, subfamily Coryphoideae, Borasseae tribe) is a massive dioecious monocot plant with its single stem reaching 30 m in height and large fan-shaped leaves spanning 1–3 m in diameter [1]. Six species are present in the Borasseae tribe including *B. aethiopum* [2], *B. akeassii* [3], *B. sambiranensis* [4] and *B. madagascariensis* [5], which are distributed in Africa, *B. heineanus* [6] found in New Guinea and *B. flabellifer,* which is solely found in Asia [1]. *B. flabellifer* is widespread in the South and Southeast Asia and is of horticultural and economic importance. The fruit is widely consumed, and the flower sap has been used for palm sugar production for hundreds of years [7]. *B. flabellifer* is currently in rapid decline due to following reasons. First, it grows extremely slow requiring 12–20 years to reach maturity and produce its first inflorescence [8]. Second, urbanization and agricultural development has eliminated a large number of the wild population [9]. Third, it reproduces via cross pollination, but there is currently no reliable mean for sex determination prior its first flowers [10]. Fourth, a clonal propagation method for this species is not well established. With these reasons, conservation and breeding programs of *B. flabellifer* is urgently needed, and genetic data are required for supporting the programs.

To date, genetic data of *B. flabellifer* are limited. A number of DNA markers including RAPD [11], ISSR [12], EST-SR and gSSR [13, 14] have been developed for studying the population in south and southeast Asia and demonstrated its low genetic diversity. However, more sequence data are still needed for detailed studies on genetic diversity and evolution. In particular, the chloroplast (cp) genome sequence would provide both species specific and population specific makers for studying *B. flabellifer.* Here, we report the complete cp genome sequence of *B. flabellifer* obtained by using both next-generation sequencing and manual gap fillings. The cp genome structure, characteristic and gene organization are described. Repetitive sequences were identified. Comparative genome analysis was performed to understand the evolutionary relationship among the Borasseae tribe.

## Main text

### The complete cp genome sequence of *B. flabellifer*

Because *B. flabellifer* leaf materials are very hard and a direct isolation of cpDNA with high purity is often difficult to obtain, chloroplast was firstly isolated using a modified protocol from Triboush et al. [15] and purified using a modified sucrose gradient method from Sandbrink et al. [16] (Additional file 1: Figure S1). The third leaf from the top (a fully expanded leaf with dark green and no more than 6-month-old) was collected and stored at 4 °C for 7 days to reduce accumulated starch before use. CpDNA was then isolated from the purified chloroplast using DNeasy Plant Mini Kit (Qiagen), and *Eco*RI restriction digests were used for verifying the purity of the cpDNA. Illumina Hiseq 2000 system generated 7,695,267 pair-end reads with an approximately 100 bp average read length. After filtering and eliminating low quality reads and contaminants using FastQC [17] and Trimmomatic [18], a total of 1,539,053,400 bp was obtained. A sliding window size of 4 with an average of Phred score ≥ 20 and removal of 5′ and 3′ ends with Phred score ≤ 3 were used as the trimming criteria. By mapping to the cp genome of *C. nucifera* (NC_022417) [19] using SOAPec v2.03, the reads provided an average of 100× sequencing depth coverage, and eight contigs covering 92% of the entire cp genome was obtained. Specific PCR amplification and sequencing were performed to fill the missing gaps. The genome map was then drawn by GenomeVx [20].

**Fig. 1:**
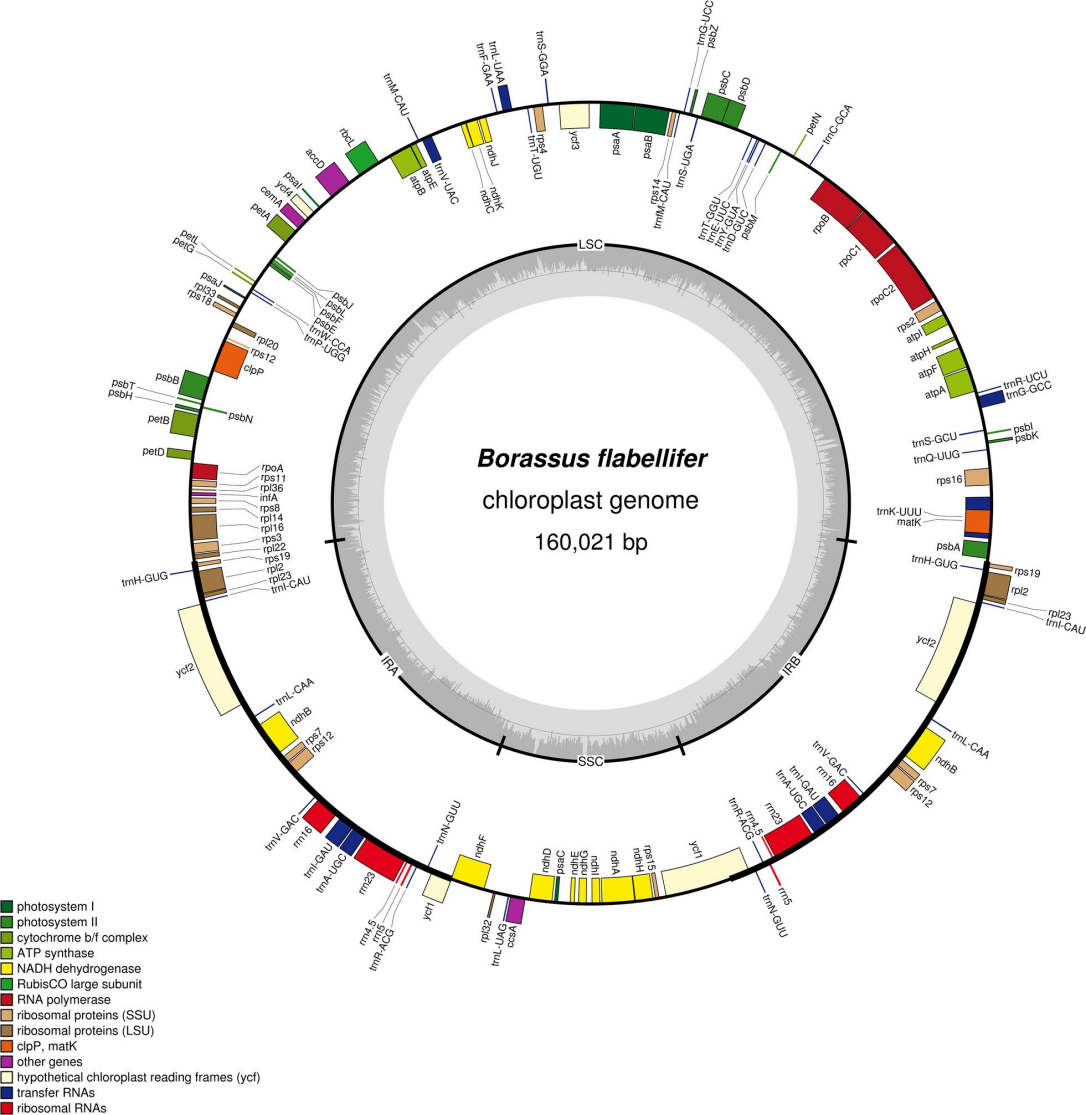
The complete chloroplast genome of *B. flabelifer*. Genes shown on the outside of the map are transcribed clockwise, and those shown on the inside are transcribed counter-clockwise. Genes functioning in related processes are coded with colors

The circular double-stranded DNA of the complete *B. flabellifer* cp genome is 160,021 bp in length (Fig. 1, GenBank Accession Number: KP_901247). It has a typical quadripartite structure composing of a pair of inverted repeat (IR) regions (27,256 bp each), a large single-copy (LCS) region (87,444 bp) and a small single-copy (SSC) region (18,065 bp). The overall GC content is 37.23%. Genome annotation using DOGMA [21] and CpGAVAS [22] with *Phoenix dactylifea* [23] as a reference and tRNAs prediction using tRNA-ScanSE [24] provided that the cp genome contains 113 unique genes: 79 protein-coding genes, 30 tRNA genes, and four rRNA genes (Additional file 2: Table S1). All of the four rRNA genes (*rrn*4.5, *rrn*5, *rrn*16 and *rrn*23), seven protein-coding genes (*rps*19, *rpl*2, *rpl*23, *ndh*B, *rps*7, *ycf*1 and *ycf* 2), two pseudogenes (*ycf*15, *ycf*68) and eight tRNA genes (*trn*H-GUG, *trn*I-CAU, *trn*L-CAA, *trn*V-GAC, *trn*I-GAU, *trn*A-UGC, *trn*R-ACG, *trn*N-GUU) are located within the IR regions. The LSC region contains 82 protein-coding genes and 21 tRNA genes, while the SSC region contains 13 protein-coding genes and one tRNA gene. The rRNA, tRNA and protein-coding genes cover 9040 bp (5.65%), 2873 bp (1.80%) and 79,368 bp (49.6%), respectively, of the complete genome.

Among 113 unique genes, there are 18 intron-containing genes (Additional file 2: Table S1): 16 genes with a single intron and two genes with two introns. Among these, *trn*K-UUU (3216 bp) contains the largest intron, in which *mat*K gene (1551 bp) is located. Four pairs of overlapped genes with different ranges of overlapped bases were observed including *atpE* and *atpB* (four overlapped bases), *ndh*K and *ndh*C (10 overlapped bases), *psb*D and *psb*C (53 overlapped bases) and *ndh*F and pseudo-*ycf*1 (60 overlapped bases). The frequency of codons in this cp genome was calculated from the exons of protein-coding genes (pseudogenes were omitted) using Maga 6 (Additional file 2: Table S2). The observed initiation codons are AUG, GUG and ACG. The GUG initiation codon was found to be specific for *rps*19 and *ndh*D, while the ACG initiation codon was found only for *rpl*2.

### Simple sequence repeats (SSRs) and repetitive sequences

Identifications of SSRs and repetitive sequences using by REPuter program (under a cut off n ≥ 10 with 100% sequence identities) [25] and GMATo v1.2 [26] showed that the cp genome contains, in total, 134 SSR loci and 47 large repeat loci (Additional file 2: Tables S3 and S4). Among the 134 SSRs, 98 and 20 loci are homopolymers and dipolymers, respectively. And, 108 loci are located in intergenic spacer (IGS) regions, while 26 loci are located in the protein-coding genes including *cem*A, *mat*K, *ndh*D, *ndh*F, *ndh*H, *rpo*C2, *rps*14, *rps*19, *rps*4 and *ycf*1. Neither pentapolymer nor hexapolymer was observed in the protein-coding regions. All 47 large repeat sequences contain four non-tandem direct repeats, six inverted repeats and 37 tandem repeats. The sizes of the repeating unit were in the range of 11–39 bp (Additional file 2: Table S4). Noting that most of the large repeats are located in the IGSs inside the single-copy regions, especially in the large single-copy region. Only eight repeats including one direct repeat and seven tandem repeats are located in the coding sequence of three protein-coding genes: *rpoC2*, *ycf1*, and *ycf2*.

### Comparative analysis of the plastid genomes among the Borasseae tribe and phylogenetic analysis among monocots

**Fig. 2:**
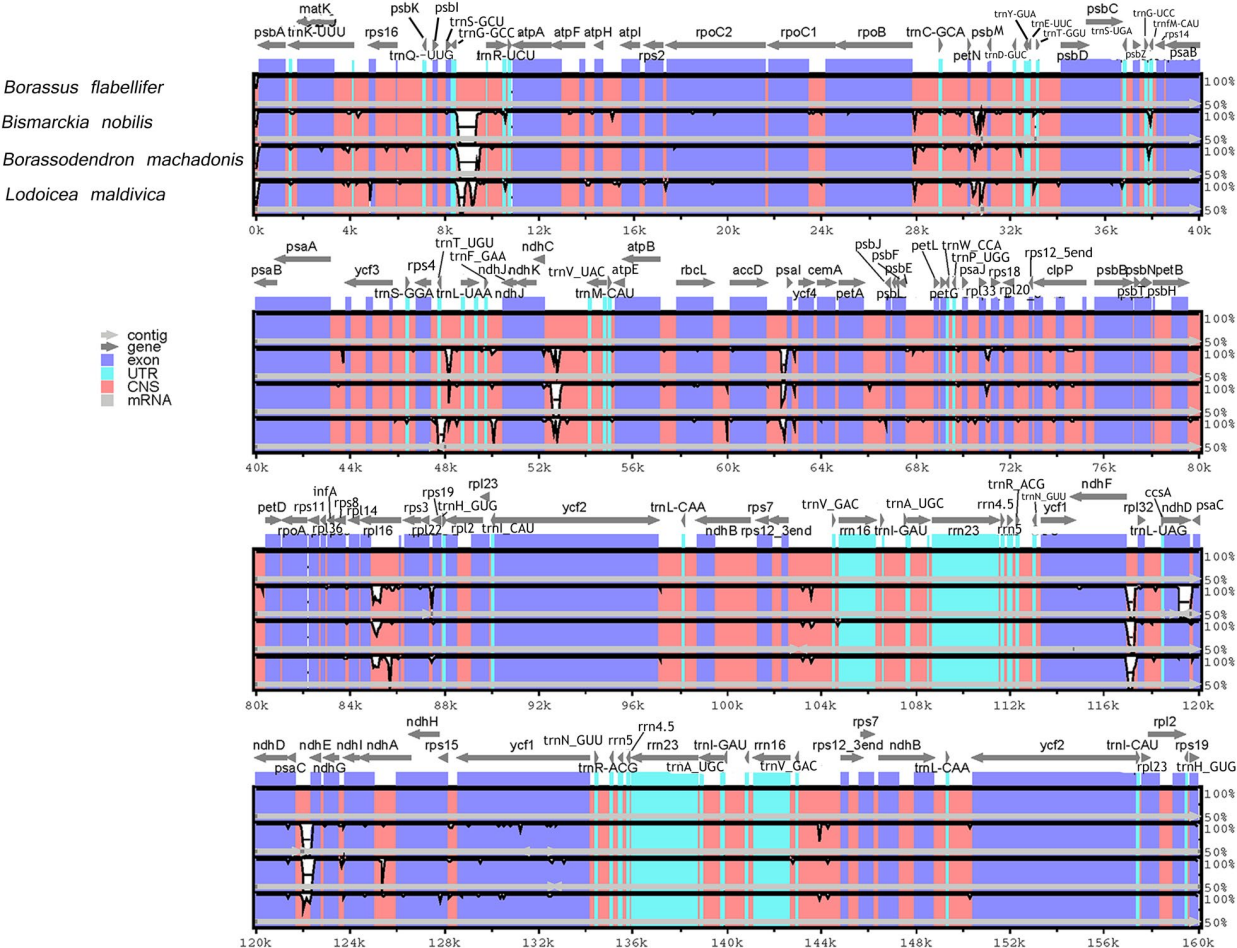
Visualization of the alignment of four Borassaseae cp genome sequences. VISTA-based identity plot shows sequence identity among four cp genomes and *B. flabellifer* cp genome as a reference. A cut-off of 70% identity was used for the plot and sequence identity is shown as a percentage between 50 and 100% on y-axis. On x-axis, *B. flabellifer* genes are indicated on top lines, and arrows represent their orientation. Genome regions are distinguished by colors. CNS indicates conserved non-coding sequences

The cp genomes of 4 species including *B. flabellifer, Bismarckia nobilis* (NC_020366.1) [27]*, Borassodendron machadonis* (NC_029969.1) [27] and *Lodoicea maldivica* (NC_029960.1) [27], which are members of Borasseae tribe are in the range between 158,144 and 160,021 bp (Additional file 2: Table S5). The differences in the cp genome sizes are due to the lengths of the LSC, SSC and IR regions. The cp genome of *B. flabellifer* is, so far, the largest among the Borasseae tribe with the longest LSC and SSC regions. These long LSC and SSC regions contain the same number of genes as in the other three cp genomes. Comparative analysis using mVISTA [28] showed that the four cp genomes are highly similar (Fig. 2). Among the four sequence regions, both IR regions are more conserved than the LSC and SSC, which contain several variable regions in the intergenic regions such as between *ndh*F-*rpl*32, *trn*G-*trn*R and *rpl32*-*trnL*. Besides, there are 11 small variable regions inside the coding regions of *acc*D*, ccsA*, *mat*K, *ndhA, ndhD, ndhF, rbcl, rpl16, rpo*C2, *rps*16 and *ycf*1. Noting that *ycf1* and *rpoC2* also carry both SSRs and large repeats.

**Fig. 3:**
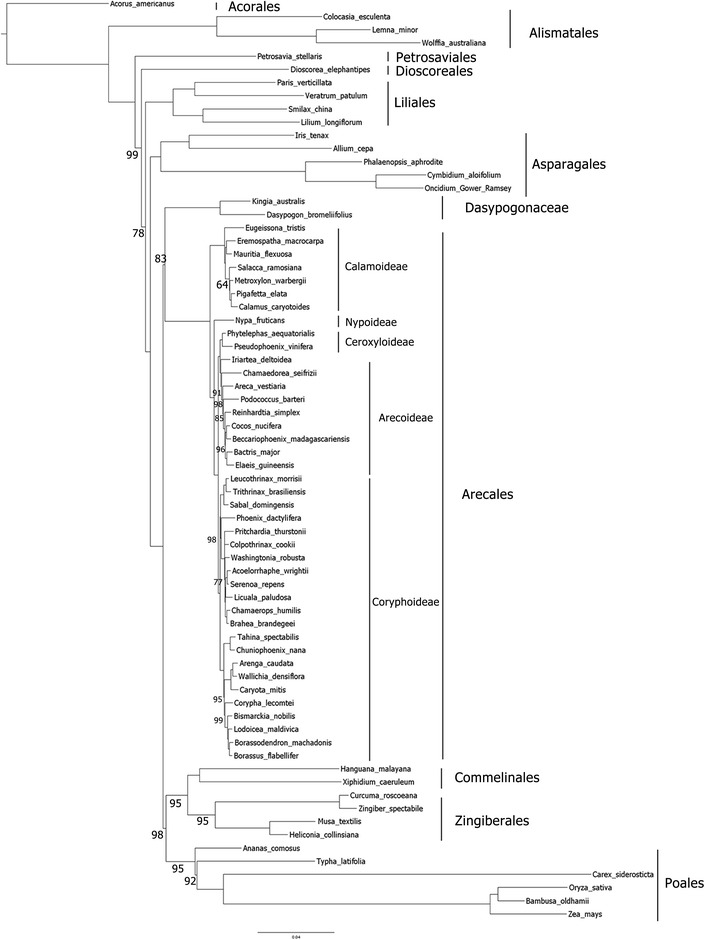
A phylogenetic tree of monocots reconstructed based on the maximum likelihood method using 74 protein-coding genes of the cp genomes. The number presented above and below each branch represents bootstrap values calculated from 1000 replicates. Those without the value indicates 100% bootstrap support

A phylogenetic tree based on the maximum likelihood method were reconstructed with raxmlGUI [29] using 74 protein-coding genes from 70 monocot species. The evolutionary relationship among monocots is presented with high bootstrap supports (Fig. 3). Previously, phylogenetic relationship among subfamilies, families and orders of the Commelinid clade using cp genomes has been described [27, 30, 31], and our result is consistent with these reports. Tribes within subfamily Coryphoideae was previously divided into two major clades: [(Phoeniceae, Livistoneae)(Sabaleae, Cryosophileae)) and (Chuniophoeniceae (Caryota (Coryphoideae, Borasseae))] [27], and, here, we provide a confirmation for this clustering with 100% bootstrap supports. The phylogenetic tree showed that *B. machadoris* is closely related to *B. flabellifer* as supported by 100% bootstrap replicates. Furthermore, our phylogenetic tree showed that the branch lengths of all members of the family Arecaceae are short, suggesting slow evolutionary rates of the cp genomes in this family.

### Limitations

The complete cp genome of *B. flabellifer* reported here provides a valuable resource for genetic analysis of this and related palm species. A number of SSRs, repetitive sequences and highly variable regions identified here would provide useful markers for studying the genetic diversity and microevolution of this species, although these have to be priory verified in a number of *B. flabellifer* populations. Indeed, further verification of these markers would provide an insight for establishing breeding and conservation programs for this palm species. Because the complete genome sequences of other plants in genus *Borassus* are not yet available, we were able to describe the evolutionary relationship among the members of Borasseae tribe, but not within the genus. Further analysis at the genus level will provide insight into recent evolutionary of palm species.

## Additional files

**Additional file 1.** A schematic procedure for isolation and purification of the chloroplast from *B. flabellifer* (a). The procedure is divided into three main steps: leaf sample preparation and disruption, chloroplast isolation and chloroplast purification. A step sucrose gradient for chloroplast purification, before and after ultra-centrifugation (b). A quality assessment of purified cpDNA using *Eco*RI digestion and agarose gel electrophoresis (c). The left panel represents cpDNA and *Eco*RI treated cpDNA isolated from chloroplast pellets without sucrose gradient purification, while the right panel represents those that isolated from chloroplast pellets with sucrose gradient purification.

**Additional file 2.** **Table S1.** Gene annotation of the *B. flabellifer* cp genome. **Table S2.** Codon usages of the *B. flabellifer* cp genome. **Table S3.** Distribution of SSRs in the *B. flabellifer* cp genome. **Table S4.** Large repeat sequences in the *B flabellifer* cp genome. **Table S5.** Comparison of the sequence sizes in four cp genomes of the Borassaseae tribe.

## Abbreviations

CP: chloroplast
IGS: intergenic spacer
IR: inverted repeat
ISSR: inter simple sequence repeat
LCS: large single-copy
RAPD: random amplified polymorphic DNA
SSC: small single-copy
SSR: short sequence repeat
