# Supplementary material for: The complete chloroplast genome sequence of Asian Palmyra palm (Borassus flabellifer)
Source: BMC Res Notes. 2017 Dec 16;10:740. doi: 10.1186/s13104-017-3077-8 (PMC5732391; doi:10.1186/s13104-017-3077-8)
Supplement: Supplementary file 2 — Additional file 2. Table S1. Gene annotation of the B. flabellifer cp genome. Table S2. Codon usages of the B. flabellifer cp genome. Table S3. Distribution of SSRs in the B. flabellifer cp genome. Table S4. Large repeat sequences in the B flabellifer cp genome. Table S5. Comparison of the sequence sizes in four cp genomes of the Borassaseae tribe. [file 13104_2017_3077_MOESM2_ESM.docx]

**Additinal file: Tables**

Supplementary Table S1. Gene annotation of the *B. flabellifer* cp genome

| Function | Gene |
| --- | --- |
| photosystemI | *psa*A, *psa*B, *psa*C, *psa*I, *psa*J, *ycf*3, *ycf*4 |
| photosystemII | *psb*A, *psb*B, *psb*C, *psb*D, *psb*E, *psb*F, *psb*H, *psb*I, *psb*J, *psb*K, *psb*L, *psb*M, *psb*N, *psb*T, *psb*Z |
| cytochrome b6/f | *pet*A, *pet*B, *pet*D, *pet*G, *pet*L, *pet*N |
| atp synthase | *atp*A, *atp*B, *atp*E, *atp*F, *atp*H, *atp*I |
| rubisco | *rbc*L |
| NADH oxidoreductase | *ndh*A, *ndh*B, *ndh*C, *ndh*D, *ndh*E, *ndh*F, *ndh*G, *ndhH*, *ndh*I, *ndh*J, *ndh*K |
| large subunit ribosomal proteins | *rpl*2, *rpl*14, *rpl*16, *rpl*20, *rpl*22, *rpl*23, *rpl*32, *rpl*33, *rpl*36 |
| small subunit ribosomal proteins | *rps*2, *rps*3, *rps*4, *rps*7, *rps*8, *rps*11, *rps*12, *rps*14, *rps*15, *rps*16, *rps*18, *rps*19 |
| RNA Protein | *rpo*A, *rpo*B, *rpo*C1, *rpo*C2 |
| other protein | *acc*D, *ccs*A, *cem*A, *clp*P, *inf*A, *mat*K |
| ribosomal RNA | *rrn*16, *rrn*23, *rrn*4.5, *rrn*5 |
| protein of unknown function | *ycf*1, *ycf*2 |
| transfer RNA | *trn*A-UGC, *trn*C-GCA, *trn*D-GUC, *trn*E-UUC, *trn*F-GAA, *trn*fM-CAU, *trn*G-GCC,*trn*G-UCC, *trn*H-GUG, *trn*I-CAU, *trnI*-GAU, *trn*K-UUU, *trn*L-CAA, *trn*L-UAA, *trn*L-UAG, *trn*M-CAU, *trn*N-GUU, *trn*P-UGG, *trn*Q-UUG, *trn*R-ACG, *trn*R-UCU, *trn*S-GCU, *trn*S-GGA, *trn*S-UGA, *trn*T-GGU, *trn*T-UGU, *trn*V-GAC, *trn*V-UAC, *trn*W-CCA, *trn*Y-GUA |
| pseudogenes | *ycf*1, *ycf*15, *ycf*68 |

Supplementary Table S2. Codon usages of the *B. flabellifer* cp genome

| Codon | Count | RSCU | Codon | Count | RSCU | Codon | Count | RSCU | Codon | Count | RSCU |
| --- | --- | --- | --- | --- | --- | --- | --- | --- | --- | --- | --- |
| UUU(F) | 933 | 1.24 | UCU(S) | 596 | 1.69 | UAU(Y) | 780 | 1.59 | UGU(C) | 250 | 1.54 |
| UUC(F) | 569 | 0.76 | UCC(S) | 351 | 1 | UAC(Y) | 200 | 0.41 | UGC(C) | 75 | 0.46 |
| UUA(L) | 806 | 1.79 | UCA(S) | 456 | 1.29 | UAA(*) | 42 | 1.47 | UGA(*) | 19 | 0.66 |
| UUG(L) | 581 | 1.29 | UCG(S) | 190 | 0.54 | UAG(*) | 25 | 0.87 | UGG(W) | 447 | 1 |
| CUU(L) | 566 | 1.25 | CCU(P) | 420 | 1.54 | CAU(H) | 506 | 1.56 | CGU(R) | 357 | 1.32 |
| CUC(L) | 200 | 0.44 | CCC(P) | 219 | 0.8 | CAC(H) | 143 | 0.44 | CGC(R) | 96 | 0.35 |
| CUA(L) | 373 | 0.83 | CCA(P) | 320 | 1.18 | CAA(Q) | 686 | 1.49 | CGA(R) | 358 | 1.32 |
| CUG(L) | 182 | 0.4 | CCG(P) | 130 | 0.48 | CAG(Q) | 237 | 0.51 | CGG(R) | 127 | 0.47 |
| AUU(I) | 1079 | 1.41 | ACU(T) | 518 | 1.52 | AAU(N) | 979 | 1.56 | AGU(S) | 415 | 1.18 |
| AUC(I) | 498 | 0.65 | ACC(T) | 251 | 0.74 | AAC(N) | 276 | 0.44 | AGC(S) | 106 | 0.3 |
| AUA(I) | 719 | 0.94 | ACA(T) | 435 | 1.28 | AAA(K) | 1035 | 1.47 | AGA(R) | 530 | 1.95 |
| AUG(M) | 628 | 1 | ACG(T) | 157 | 0.46 | AAG(K) | 371 | 0.53 | AGG(R) | 159 | 0.59 |
| GUU(V) | 514 | 1.42 | GCU(A) | 612 | 1.82 | GAU(D) | 875 | 1.61 | GGU(G) | 601 | 1.36 |
| GUC(V) | 189 | 0.52 | GCC(A) | 207 | 0.62 | GAC(D) | 211 | 0.39 | GGC(G) | 152 | 0.34 |
| GUA(V) | 546 | 1.51 | GCA(A) | 383 | 1.14 | GAA(E) | 1054 | 1.5 | GGA(G) | 730 | 1.65 |
| GUG(V) | 200 | 0.55 | GCG(A) | 143 | 0.43 | GAG(E) | 353 | 0.5 | GGG(G) | 290 | 0.65 |

Supplementary Table S3. Distribution of SSRs in the *B. flabellifer* cp genome

| Type | | Repeat | Location | Total numbers of SSRs |
| --- | --- | --- | --- | --- |
|  |  |  |  |  |
| mono | A | 9 | 20-IGS,2-ycf1,1-rps19 | 23 |
|  |  | 10 | 9-IGS,2-ycf1 | 11 |
|  |  | 11 | 2-IGS,1-cemA,1-ycf1 | 4 |
|  |  | 13 | 6-IGS | 6 |
|  |  | 15 | 1/1-IGS | 1 |
|  | T | 9 | 20-IGS,1-matK,1-rps19,4-ycf1 | 26 |
|  |  | 10 | 11-IGS,1-1-rpoC2 | 12 |
|  |  | 11 | 2-IGS,1-rpoC2,1-rps14,1-ycf1 | 5 |
|  |  | 12 | 3-IGS | 3 |
|  |  | 13 | 1-IGS,1-ycf1 | 2 |
|  |  | 15 | 1-IGS | 1 |
|  |  | 17 | 1-IGS | 1 |
|  | C | 9 | 1-IGS,1-ndhF | 2 |
|  | G | 9 | 1-rps4 | 1 |
| di | AT | 5 | 5-IGS,1-rpoC2 | 6 |
|  |  | 6 | 2-IGS | 2 |
|  |  | 7 | 2-IGS | 2 |
|  |  | 8 | 2-IGS | 2 |
|  | TA | 5 | 3-IGS | 3 |
|  |  | 6 | 2-IGS | 2 |
|  |  | 7 | 1-IGS | 1 |
|  | TC | 5 | 1-cemA,1-ndhH | 2 |
| tri | AAT | 5 | 1-IGS | 1 |
|  | ACT | 4 | 1-matK | 1 |
| tetra | ATTT | 3 | 1-IGS | 1 |
|  | ATTC | 3 | 1-IGS | 1 |
|  | ATAA | 3 | 1-IGS | 1 |
|  | AATA | 3 | 1-ndhD | 1 |
|  | AATG | 3 | 1-cemA | 1 |
|  | TATT | 3 | 1-IGS | 1 |
|  | TCTA | 4 | 1-IGS | 1 |
| penta | AAATA | 3 | 1-IGS | 1 |
|  | ATGTA | 3 | 1-IGS | 1 |
|  | CAAAT | 3 | 1-IGS | 1 |
|  | TATTT | 3 | 1-IGS | 1 |
|  | TTTCA | 3 | 1-IGS | 1 |
| hexa | ACTAAT | 9 | 1-IGS | 1 |
|  | TATTAG | 9 | 1-IGS | 1 |

Supplementary Table S4. Large repeat sequences in the *B flabellifer* cp genome.

| No | Size (bp) | Repeat sequences | Type | Region |
| --- | --- | --- | --- | --- |
| 1 | 17 | AATGGAGCAATACCCAA | I | LSC; IGS rps19-psbA |
| 2 | 12 | ATCCTGAATGTG | T | LSC; IGS rps16-trnQ-UUG |
| 3 | 23 | ATAAAATATATTATAAAGGAAAT | T | LSC; IGS trnS-GCU-trnG-GCC |
| 4 | 12 | TATGTACATATA | T | LSC; IGS trnS-GCU-trnG-GCC |
| 5 | 17 | TTCTTTATTTGTATTTG | T | LSC; intron of atpF gene |
| 6 | 14 | TGTCTCATGGAAAT | D | LSC; CDS rpoC2 gene |
| 7 | 21 | CCATATAATATATGTAATGTA | T | LSC; IGS rpoB-trnC-GCA |
| 8 | 11 | TTTTATTTTAT | T | LSC; IGS petN-psbM |
| 9 | 23 | TAGTGTGGTAGAAAGAACTATAT | I | LSC; IGS petN-psbM |
| 10 | 18 | ATCTCGGGAC | T | LSC; IGS petN-petM |
| 11 | 11 | ACATATATGCA | D | LSC; IGS trnE-UUC-trnT-GGU |
| 12 | 15 | AATTCGATATAGATT | T | LSC; IGS trnT-GGU-psbD |
| 13 | 13 | TATTATTATCAAT | T | LSC; IGS rps14-psaB |
| 14 | 26 | ATTTATCCCTATGAGATACCATACTA | T | LSC; IGS psaA-ycf3 |
| 15 | 12 | ATTTCATCTAAG | T | LSC; IGS ycf3-trnS-GGA |
| 16 | 18 | TCTATATTATTAGTTATA | I | LSC; IGS trnT-UGU-trnL-UAA |
| 17 | 15 | TATTTTTCTAATAGA | T | LSC; IGS trnT-UGU-trnL-UAA |
| 18 | 15 | TATATGACTAATTAG | T | LSC; IGS trnT-UGU-trnL-UAA |
| 19 | 14 | ATTTATACATTCTA | T | LSC; IGS trnT-UGU-trnL-UAA |
| 20 | 12 | CCATTTTACTTC | T | LSC; IGS trnL-UAA-trnF-GAA |
| 21 | 20 | AAGTAATTAACAAGTCAAAT | T | LSC; IGS ndhC-trnV-UAC |
| 22 | 26 | ATAATATATAATGAGATAATGAAAAT | D | LSC; IGS ndhC-trnV-UAC |
| 23 | 11 | AAAAAATATAA | T | LSC; IGS ndhC-trnV-UAC |
| 24 | 11 | ATATAATTAAT | T | LSC; IGS n dhC-trnV-UAC |
| 25 | 11 | CATTATCTCTG | T | LSC; IGS ndhC-trnV-UAC |
| 26 | 16 | AACTCAATACTTCTAT | T | LSC; IGS rbcL-accD |
| 27 | 16 | TTATTAACTACTTAT | D | LSC; IGS accD-psaI |
| 28 | 11 | TTATAAATATT | T | LSC; IGS accD-psaI |
| 29 | 39 | TATGCATGCGGATACATGAT | T | LSC; IGS psaI-ycf4 |
|  |  | ATCCGCATAAATGCATGTA |  |  |
| 30 | 25 | AGTAAGAACTCAGCGGGACCTTACT | I | LSC; IGS petA-psbJ |
| 31 | 14 | AATAAATATACTAT | T | LSC; IGS psbE-petL |
| 32 | 14 | ATTTATTGATATTT | T | LSC; intron of rpl16 gene |
| 33 | 20 | CTCGTTTACAAATATCCAAA | T | LSC; IGS rpl16 -rps3 |
| 34 | 12 | ATTGAGAGAGAT | T | Ira; CDS ycf2 gene |
| 35 | 15 | TTCTTCCTATACCTA | T | Ira; CDS ycf2 gene |
| 36 | 24 | GATATCGATATTGATGATAGTGAC | T | Ira; CDS ycf2 gene |
| 37 | 17 | ATTATTTGTTATTTTAT | T | SSC; IGS ndhF-rpl32 |
| 38 | 19 | ATTTATTTGATTTTTTTCA | I | SSC; IGS rpl32-trnL-UAG |
| 39 | 20 | TTCGAGTTTTTGTTTTGACA | I | SSC; IGS ccsA-ndhD |
| 40 | 13 | ATTCAATATGAAA | T | SSC; IGS psaC-ndhE |
| 41 | 19 | ATTATTCAATAGTAAATTA | T | SSC; IGS psaC-ndhE |
| 42 | 12 | TTATATAAGATA | T | SSC; IGS psaC-ndhE |
| 43 | 11 | TAAAAACCTTA | T | SSC; IGS ndhE-ndhG |
| 44 | 12 | TTTTCTAATTTT | T | SSC; CDS ycf1 gene |
| 45 | 24 | ATATCGTCACTATCATCAATATCG | T | Irb; CDS ycf2 gene |
| 46 | 15 | ATAGGTATAGGAAGA | T | Irb; CDS ycf2 gene |
| 47 | 12 | ATCTCTCTCAAT | T | Irb; CDS ycf2 gene |

Supplementary Table S5. Comparison of the sequence sizes in four cp genomes of the Borassaseae tribe

|  | Accession Number | Size (bp) | LSC (bp) | SSC (bp) | Irs (bp) |
| --- | --- | --- | --- | --- | --- |
| *B. flabellifer* | KP901247 | 160,021 | 87,444 | 18,065 | 54,512 |
| *B. nobilis* | NC_020366.1 | 158,211 | 86,358 | 17,459 | 54,364 |
| *B. machadonis* | NC_029969.1 | 158,144 | 86,139 | 17,397 | 54,608 |
| *L. maldivica* | NC_029960.1 | 159,010 | 86,562 | 17,876 | 54,572 |
